# The Effects of Inlet Blockage and Electrical Driving Mode on the Performance of a Needle-Ring Ionic Wind Pump

**DOI:** 10.3390/mi12080900

**Published:** 2021-07-29

**Authors:** Jia-Cheng Ye, Tsrong-Yi Wen

**Affiliations:** Department of Mechanical Engineering, High Speed 3D Printing Research Center, National Taiwan University of Science and Technology, E1-441, No 43, Sec 4, Keelung Rd, Taipei 106, Taiwan; m10803317@mail.ntust.edu.tw

**Keywords:** electrohydrodynamic, needle-ring, ionic wind pump, inlet blockage

## Abstract

The thermal management of microelectronics is important because overheating can lead to various reliability issues. The most common thermal solution used in microelectronics is forced convection, which is usually initiated and sustained by an airflow generator, such as rotary fans. However, traditional rotary fans might not be appropriate for microelectronics due to the space limit. The form factor of an ionic wind pump can be small and, thus, could play a role in the thermal management of microelectronics. This paper presents how the performance of a needle-ring ionic wind pump responds to inlet blockage in different electrical driving modes (direct current), including the flow rate, the corona power, and the energy efficiency. The results show that the performance of small needle-ring ionic wind pumps is sensitive to neither the inlet blockage nor the electrical driving mode, making needle-ring ionic wind pumps a viable option for microelectronics. On the other hand, it is preferable to drive needle-ring ionic wind pumps by a constant current if consistent performance is desired.

## 1. Introduction

Thermal management for microelectronics is receiving more and more attention because overheating can lead to significant failures [1,2,3]. Forced convection is an effective strategy used in thermal management. Rotary fans are the most common devices used to initiate and sustain the required airflow in forced convection. However, a traditional rotary fan consists of several components (motors, blades, etc.) that are volume demanding. Additionally, there is a no-flow region at the immediate outlet of a rotary fan because of the motor (hub) [4,5]. On the other hand, it is known that the efficiency of a small rotary fan can be tremendously low [6]. Thus, rotary fans are not favorable for the thermal management of microelectronics.

**Figure 1:**
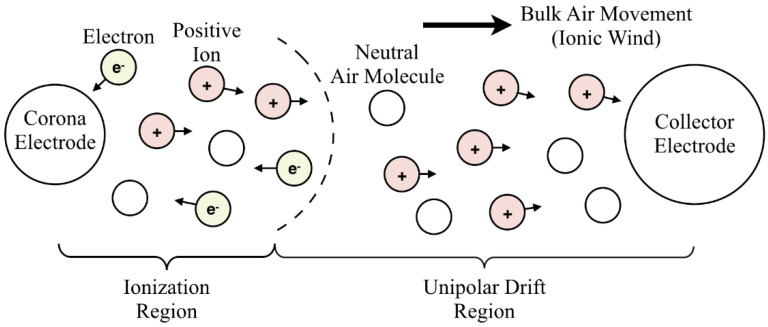
The generation of positive ionic wind. Reproduced with permission from [15].

Ionic wind pumps are airflow generators that work based on electrohydrodynamics (EHDs), coupling the electrostatics, the fluid mechanics, and the charge transport [7,8,9]. Figure 1 shows the schematic of positive ionic wind generation. When an appropriate high electric field (potential difference) is applied between the corona electrode and the collector electrode, the ionization region and the unipolar drift region will be formed in between because of the corona discharge. Air molecules will be charged when passing around the ionization region. The charged air molecules will then move to the collector electrode following the electric field, transferring charges and momentum to other air molecules [10]. The bulk air movement is the result, also known as ionic wind or EHD flow [11,12]. Because there are no moving parts required, ionic wind pumps are noise free, vibration free, and the form factor can be small [13,14]. Unlike traditional rotary fans, the electrode configuration of an ionic wind pump can be flexible such that the no-flow region can be avoided, which is highly desirable for compact microelectronics.

For an ionic wind pump, the corona electrode can be something that has a high curvature, such as a thin wire and a needle, while the collector electrode usually has a low curvature, such as a thick cylinder, plate, and ring [16,17,18,19]. Among various electrodes, this paper focuses on needle-ring ionic wind pumps because of their high flexibility in the number and configuration of the needles [17,20,21,22,23] and, thus, high applicability [24,25,26,27,28]. On the other hand, the performance of an air pump is often represented by the curves of the pressure against the flow rate (PQ curves) at a free inlet condition. However, when coming to practice, the pump inlet could have obstacles, such as the components or chassis, creating negative effects on the PQ performance, known as the inlet blockage effect [29,30,31]. The PQ performance of a wire-rod ionic wind pump has been shown to be affected by both the inlet blockage and the electrical driving mode [15,32].

Yet, to date, no blockage-induced performance degradation for needle-ring ionic wind pumps has been reported. To better estimate the performance when applying needle-ring ionic wind pumps in practice, this paper presents and discusses the characteristic changes of the flow rate, the corona power, and the energy efficiency with respect to the inlet blockage, the electrical driving mode, and the pump size.

## 2. Material and Methods

### 2.1. Experimental Setup

**Figure 2:**
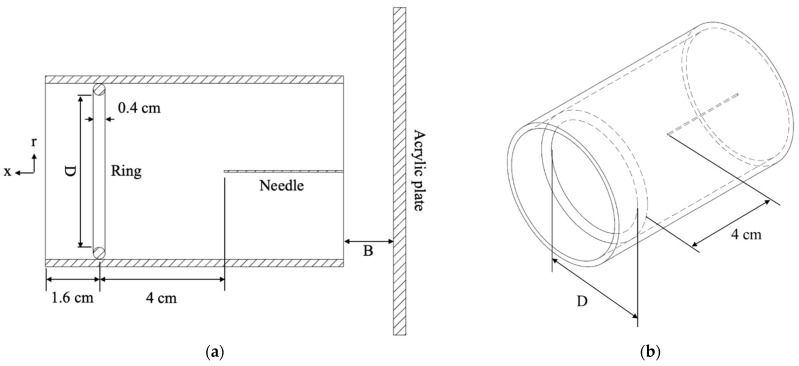
The schematics of the experimental setup and the ionic wind pump under test. (**a**) Experimental setup (center cut-plane view) and (**b**) ionic wind pump (iso view). D represents the pump size (inner diameter of the ring), and B represents the blockage distance.

Figure 2a shows the schematic of the experiment setup. An acrylic plate of 80 cm by 80 cm was placed upstream of the inlet of the ionic wind pump under test in a center-to-center manner, presenting as the inlet blockage. The blockage distance (acrylic plate to pump inlet) was a parameter of interest, which was set to be 1 cm, 2 cm, 3 cm, and infinity (no blockage). A hotwire anemometer (Testo 405V1) was placed 0.1 cm away from the outlet of the ionic wind pump under test to measure the ionic wind velocity and, thus, the flow rate (5-point at r = 0, ±0.2D, and ±0.4D). Every measurement was repeated three times to ensure repeatability.

### 2.2. Ionic Wind Pump under Test

The ionic wind pump was a single needle-to-ring configuration, as shown in Figure 2b. The needle was made of stainless steel and had a diameter of 0.06 cm. The ring was also made of stainless steel and had a cross-sectional diameter of 0.4 cm. The distance from the needle tip to the ring center was 4 cm. The pump size was characterized by the inner diameter of the ring (D) and was a parameter of interest, which was taken to be 3 cm, 5 cm, and 10 cm. The needle was applied at a high voltage, and the ring was electrically grounded. The direct current (DC) high-voltage power supply (Matsusada AU-40P2.5-LCF, Shiga, Japan) used here provided two corona operating modes to the needle, which were constant voltage (C.V.) and constant current (C.C.).

## 3. Results and Discussion

### 3.1. Flow Rate

**Figure 3:**
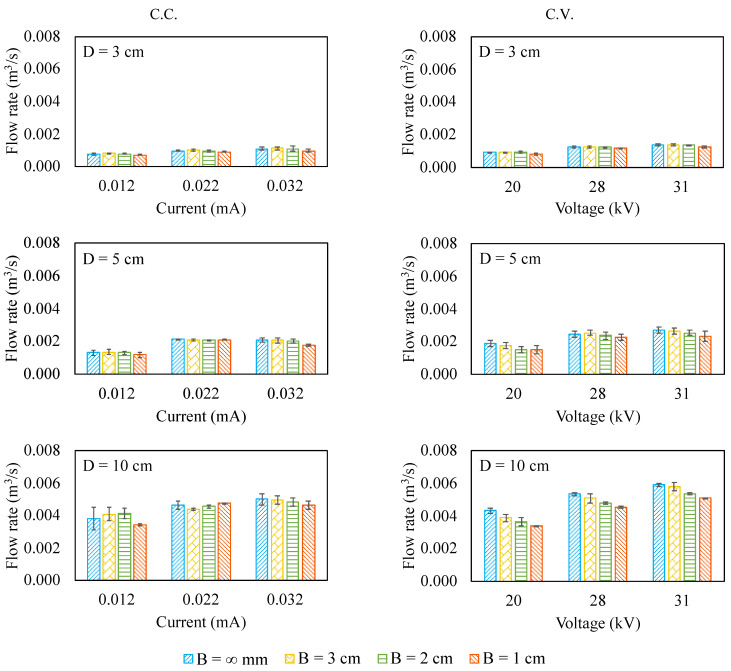
The flow rate for the ionic wind pump under test.

Figure 3 shows the flow rates for the cases when operating under either C.C. or C.V. mode. It can be seen that the flow rate is proportional to the corona voltage/current and the pump size, as expected. The flow rate is the product of velocity and the cross-sectional area. The cross-sectional area of the pump increases when the pump size increases. However, under the same electrical conditions, the ionic wind velocity is inversely proportional to the pump size because the resultant electric field strength decreases when the pump size increases (the needle-to-ring distance increases). Among these pumps under test, the decreasing rate of the ionic wind velocity is lower than the increasing rate of the cross-sectional area when the pump size increases. As a result, the flow rate still increases over the pump size.

The influence of the inlet blockage on the flow rate is not obvious for the 3 cm and 5 cm pumps regardless of the electrical driving mode. Nonetheless, for the 10 cm pump, the inlet blockage, the electrical driving mode, and the flow rate do have connections. When driving such a pump in C.C. mode, the flow rate does not change a lot when the inlet blockage presents. However, when driving such a pump in C.V. mode, the inlet blockage can lower the flow rate by 13.6% (for 31 kV) to 20.1% (for 20 kV). This phenomenon is different from what has been reported for the wire-rod ionic wind pump [15]. The suspected reason for the difference between these two types of ionic wind pumps is that the distributions of the space charge density around the corona electrode and the inlet velocity profile are different. This reflects the fact that the electrode configuration plays a meaningful role for ionic wind pumps with regard to resisting the inlet blockage.

### 3.2. Corona Power

**Figure 4:**
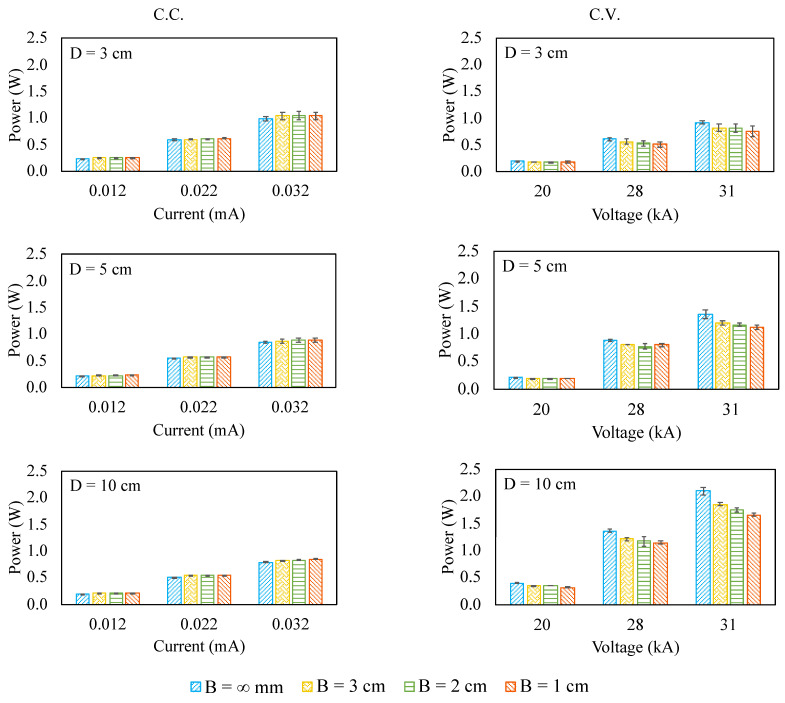
The corona power for the ionic wind pump under test.

Figure 4 shows the corona power of the ionic wind pump under test. Undoubtedly, the corona power is proportional to the applied voltage or current. The response of the corona power to the inlet blockage is dramatically different in terms of the electrical driving mode. When it is in C.C. mode, the corona power increases over the inlet blockage, even just at a somewhat low rate. In other words, the corona voltage becomes a little higher when the inlet blockage gets closer to the pump. However, when it is in C.V. mode, the corona power decreases over the inlet blockage. In other words, the corona current becomes lower when the inlet blockage gets closer to the pump. Such a characteristic is one of the reasons that driving an ionic wind pump in C.C. mode better withstands the inlet blockage than that in C.V. mode. This unique behavior could be attributed to the characteristics of the current density, which is a function of both the electric field and the airflow field (velocity), as shown in Equation (1) [11,33]. Under C.C. mode (i.e., the current density is a constant), the airflow velocity and the electric field are competing. When the airflow velocity decreases, the electric field should increase in order to make the current density constant. Thus, the voltage increases so that the corona power increases. On the contrary, under C.V. mode (i.e., the electric field is a constant), the current density is proportional to the airflow velocity. When the airflow velocity decreases, the current density should decrease. As a result, the current decreases and so does the corona power.

(1) J=ρ(μE+w)−D∇ρ

where J is the current density, ρ is the space charge density, μ is the ion mobility, E is the electric field, is the airflow field (velocity), and D is the diffusion coefficient.

### 3.3. Energy Efficiency

**Figure 5:**
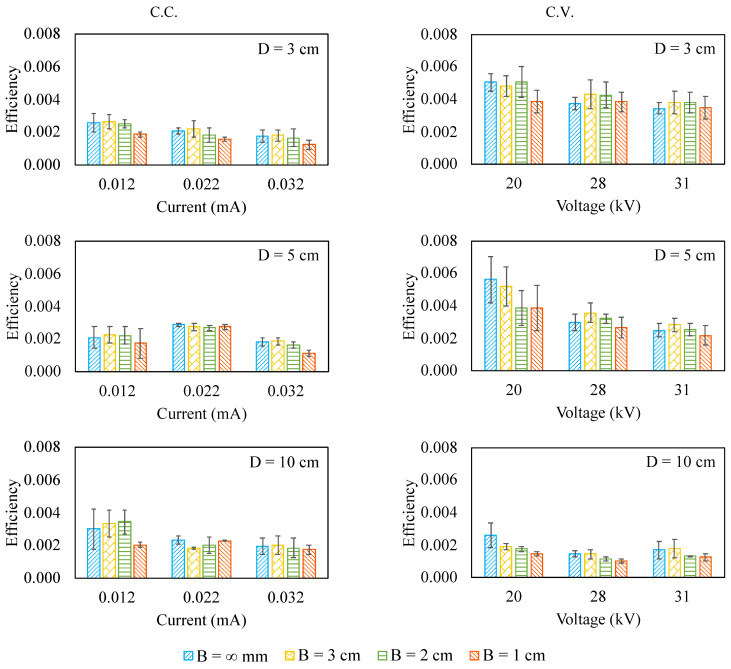
The energy efficiency for the ionic wind pump under test.

The energy efficiency is defined by the ratio of the airflow power to corona power (electric power input) as shown in Equation (2), and the results are shown in Figure 5.

(2) $$\eta =\frac{\mathrm{Airflow}\;\mathrm{Power}}{\mathrm{Corona}\;\mathrm{Power}}=\frac{\frac{1}{2}\times \dot{m}\times V^{2}}{I\times V_{e}}$$

where η is the energy efficiency, $\dot{m}$ is the mass flow rate, V is the airflow velocity, I is the corona current, and $V_{e}$ is the corona voltage.

For the 3 cm and 5 cm pumps, the energy efficiency seems to fluctuate over the inlet blockage regardless of the electrical driving mode. On the other hand, for the 10 cm pump driven by C.V. mode, the energy efficiency has a clear decrease when the inlet blockage presents. As for the 10 cm pump driven by C.C. mode, the energy efficiency fluctuates and does not have a consistent increasing or decreasing trend.

Furthermore, it is also interesting to realize that the energy efficiency tends to decrease when the corona current or corona voltage increases, coincident with what has been reported in the literature [12,34]. It is simply because the increase in the corona power outruns that in airflow power, lowering the energy efficiency.

Moreover, when it is in C.C. mode, the pump size does not seem to have a significant effect on the energy efficiency. However, when it is in C.V. mode, the 3 cm pump outperforms the 10 cm one in energy efficiency. This makes sense because the airflow can be generated at a lower voltage for a smaller pump (lower onset voltage), making the ratio of the airflow power to the corona power larger.

## 4. Conclusions

This paper presents the flow rate, the corona power, and the energy efficiency of the needle-ring ionic wind pump in terms of the inlet blockage, the pump size, and the electrical driving mode. The results indicate that driving a big needle-ring ionic wind pump by a constant current results in a more consistent airflow rate with respect to the inlet blockage when compared with that by constant voltage. Under C.C. mode, the corona power increases over the inlet blockage. However, under C.V. mode, the corona power decreases over the inlet blockage. This is also the reason why the airflow rate changes a little over the inlet blockage when the ionic wind pump is driven by C.C. mode. Regarding energy efficiency, the small needle-ring ionic wind pump has a higher energy efficiency than the big one has, which is in contrast to what traditional rotary fans have. Moreover, the energy efficiency of such a small needle-ring ionic wind pump is not sensitive to both the electrical driving mode and the inlet blockage. All these characteristics make needle-ring ionic wind pumps a viable option for applications that have space limitations and require consistent performance.

## Data Availability

Not applicable.

## References

[1] Akbari S., Lovberg A., Tegehall P.E., Brinkfeldt K., Andersson D. (2019). Effect of PCB cracks on thermal cycling reliability of passive microelectronic components with single-grained solder joints. Microelectron. Reliab..

[2] Ekpu M. (2021). Investigating the reliability of SnAgCu solder alloys at elevated temperatures in microelectronic applications. J. Electron. Mater..

[3] Lall P., Pecht M.G., Hakim E.B. (2020). Influence of temperature on microelectronics and system reliability: A physics of failure approach.

[4] Belarbi A.A., Beriache M., Bettahar A. (2018). Experimental study of aero-thermal heat sink performances subjected to impinging air flow. Int. J. Heat Technol..

[5] Shankaran G.V., Dogruoz M.B. Validation of an advanced fan model with multiple reference frame approach. Proceedings of the 12th IEEE Intersociety Conference on Thermal and Thermomechanical Phenomena in Electronic Systems.

[6] Mathson T., Ivanovich M. (2011). AMCA’s fan efficiency grades: Answers to frequently asked questions. AMCA Int..

[7] Johnson M.J., Go D.B. (2017). Recent advances in electrohydrodynamic pumps operated by ionic winds: A review. Plasma Sources Sci. Technol..

[8] Moreau E., Audier P., Benard N. (2018). Ionic wind produced by positive and negative corona discharges in air. J. Electrost..

[9] Wang H.C., Jewell-Larsen N.E., Mamishev A.V. (2013). Thermal management of microelectronics with electrostatic fluid accelerators. Appl. Therm. Eng..

[10] Wen T.Y., Lindgren K., Mamishev A.V. (2016). Electrostatic fluid accelerator under high-speed free air stream. Contrib. Plasma Phys..

[11] Zhao L., Adamiak K. (2005). EHD flow in air produced by electric corona discharge in pin-plate configuration. J. Electrost..

[12] June M.S., Kribs J., Lyons K.M. (2011). Measuring efficiency of positive and negative ionic wind devices for comparison to fans and blowers. J. Electrost..

[13] Kawamoto H., Umezu S. (2008). Electrostatic micro-ozone fan that utilizes ionic wind induced in pin-to-plate corona discharge system. J. Electrost..

[14] Ong A.O., Abramson A.R., Tien N.C. (2014). Electrohydrodynamic microfabricated ionic wind pumps for thermal management applications. J. Heat Transf..

[15] Wang P.H., Wen T.Y. (2020). Effects of inlet blockage and electrical driving mode on static pressure and flow rate of wire-rod electrohydrodynamic pumps. J. Electrost..

[16] Colas D.F., Ferret A., Pai D.Z., Lacoste D.A., Laux C.O. (2010). Ionic wind generation by a wire-cylinder-plate corona discharge in air at atmospheric pressure. J. Appl. Phys..

[17] Moon J.D., Hwang D.H., Geum S.T. (2009). An EHD gas pump utilizing a ring/needle electrode. IEEE Trans. Dielectr. Electr. Insul..

[18] Ongkodjojo A., Roberts R.C., Abramson A.R., Tien N.C. Highly efficient ionic wind-based cooling microfabricated device for microchip cooling applications. Proceedings of the Solid-State Sensors, Actuators, and Microsystems Workshop.

[19] Defoort E., Benard N., Moreau E. (2017). Ionic wind produced by an electro-aerodynamic pump based on corona and dielectric barrier discharges. J. Electrost..

[20] Zhang J., Kong L., Qu J., Wang S., Qu Z. (2019). Numerical and experimental investigation on configuration optimization of the large-size ionic wind pump. Energy.

[21] Birhane Y.T., Lin S.C., Lai F.C. (2015). Flow characteristics of a single stage EHD gas pump in circular tube. J. Electrost..

[22] Birhane Y.T., Lin S.C., Lai F.C. (2017). Flow characteristics of a two-stage EHD gas pump in a circular pipe. IEEE Trans. Ind. Appl..

[23] Zhang Y., Liu L., Chen Y., Ouyang J. (2015). Characteristics of ionic wind in needle-to-ring corona discharge. J. Electrost..

[24] Qu J., Zhang J., Li M., Tao W. (2020). Heat dissipation of electronic components by ionic wind from multi-needle electrodes discharge: Experimental and multi-physical analysis. Int. J. Heat Mass Transf..

[25] Wang S., Qu J.G., Kong L.J., Zhang J.F., Qu Z.G. (2019). Numerical and experimental study of heat-transfer characteristics of needle-to-ring-type ionic wind generator for heated-plate cooling. Int. J. Therm. Sci..

[26] Kawamoto H., Ichikawa R. (2021). Development of ionic wind pump used in martian environment. J. Aerosp. Eng..

[27] Xu C., Zheng H., Liu J., Chu J., Zeng X., Sun R., Liu S. (2020). Enhanced cooling of LED filament bulbs using an embedded tri-needle/ring ionic wind device. Energies.

[28] Lee S.J., Li L., Kwon K., Kim W., Kim D. (2015). Parallel integration of ionic wind generators on PCBs for enhancing flow rate. Microsyst. Technol..

[29] Lin S.C., Chou C.A. (2004). Blockage effect of axial-flow fans applied on heat sink assembly. Appl. Therm. Eng..

[30] Wang C. (2018). Noise source analysis for two identical small axial-flow fans in series under operating condition. Appl. Acoust..

[31] Fukue T., Koizumi K., Ishizuka M., Nakagawa S. (2011). Effects of electronic enclosure and inlet area on P-Q curves of installed axial cooling fans. J. Environ. Eng..

[32] Wang P.H., Wen T.Y. (2020). Effects of electrical driving mode on pressure and flow rate of wire-rod electrohydrodynamic pumps. IEEE Trans. Compon. Packag. Manuf. Technol..

[33] Marciulionis P. (2020). Analysis of electrohydrodynamic air flow induced by DC corona field in wire-to-plane electrode system. J. Electrost..

[34] Rickard M., Dunn-Rankin D. (2007). Numerical simulation of a tubular ion-driven wind generator. J. Electrost..

